# Acceptability Among Community Healthcare Nurses of Intelligent Wireless Sensor-system Technology for the Rapid Detection of Health Issues in Home-dwelling Older Adults

**DOI:** 10.2174/1874434601711010054

**Published:** 2017-04-17

**Authors:** Christine Cohen, Thomas Kampel, Henk Verloo

**Affiliations:** 1 Univeristy of Applied Sciences of Western Switzerland, La Source, Avenue Vinet 30, CH – 1004, Lausanne, Switzerland; 2 Univeristy of Applied Sciences of Western Switzerland, Departement Nursing Sciences, Chemin de l'Agasse, 6, CH - 1951, Sion, Switzerland.

**Keywords:** Acceptability, Community healthcare nurses, Qualitative research, Gerontechnology, Home-dwelling older adults, Implementation, Intelligent wireless sensor systems, Perceived usefulness, Perceived ease of use, Satisfaction

## Abstract

**Background:**

The effective care and support of community healthcare nurses (CHNs) contribute greatly to the healthy aging of older adults living at home. Integrating innovative technologies into CHNs’ daily practice offers new opportunities and perspectives for early detection of health issues and interventions among home-dwelling older adults.

**Aim:**

To explore the perception of acceptability among CHNs of an intelligent wireless sensor system (IWSS) for use in daily practice for the detection of health issues in home-dwelling older adults receiving home healthcare.

**Method:**

Descriptive and qualitative data were sourced from a pilot randomized controlled trial involving 17 CHNs using an IWSS in their daily practice to rapidly detect falls and other health issues in patients’ homes. IWSS alerts indicating behavior changes were sent to CHNs. Their perceived usefulness (PU) and perceived ease of use (PEOU) were assessed. The acceptability of IWSS technology was explored using a questionnaire and focus group discussions.

**Results:**

The PU and PEOU of the IWSS technology were low to moderate. A majority of the CHNs were dissatisfied with its performance and intrusiveness; they reported multiple obstacles in the usefulness and ease of use of the IWSS technology in daily practice.

**Conclusion:**

To improve the IWSS technology’s low to moderate acceptability among CHNs, we recommend a more user-centered implementation strategy and an embedded model of nursing care.

## INTRODUCTION

By 2020, Switzerland’s population will have about 1.2 million older adults [1]. The majority of home-dwelling older adults wish to live in their own homes for as long as possible, even when they have significant healthcare needs [2]. Extremely rapid, innovative technological development is occurring in parallel to this substantial demographic transformation [3]. These emerging technologies could help community healthcare nurses (CHNs) to continuously monitor the health status of home-dwelling older adults facing physical and cognitive decline and enable them to remain safely in their homes [4, 5]. Embedded sensor devices and innovative technology can clearly help nurses detect health issues early [6]. They could also be useful for evaluating nursing interventions among home-dwelling older adults that are aimed at preventing acute physical decline and monitoring chronic diseases, with the ultimate goal of keeping them in their homes longer [7]. However, Rantz *et al.* (2015) indicated that no evidence existed that sensors were more effective than usual care in a nursing care coordination model [7]. This new type of intervention strategy could reinforce and preserve independence and quality of life, thus enabling this vulnerable group to remain at home longer, even if they have moderate neurocognitive impairment [8].

Thus, CHNs using the benefits of advanced technology in their daily practice could promote the preferred form of care of both older patients and public health policymakers [9]. Different types of healthcare providers – but especially CHNs – are exploring alternative means of offering new high-performance services for maintaining older adults in deteriorating health at home and improving their quality of life [10]. However, to better predict how acceptable and useful innovative technologies might be, it is important to understand the factors that influence their acceptance and use in daily practice, especially among the CHNs using them directly. Several studies have demonstrated the influence of perceived usefulness (PU), perceived ease of use (PEOU) and other relevant determinants of the acceptance and use of innovative technologies [11, 12]. Previous acceptance research has been criticized for being too reliant on effectiveness and the proof of concept approach, overlooking such essential determinants as the device’s usefulness, ease of use and ease to implement [13-15]. Furthermore, most acceptance research has focused on communication and assistive technology for older adults in the home, neglecting other types of technology [16]. These concerns indicate that more research is needed to develop a better understanding of the acceptance of various types of technology useful in daily practice, not only acceptance by home-dwelling older adults but also by the CHNs caring for them. This article describes how CHNs evaluated the acceptability of an innovative intelligent wireless sensor-system (IWSS) for rapidly detecting health issues among home-dwelling older adults in daily practice.

## THEORETICAL FRAMEWORK

The present study used the Technology Acceptance Model (TAM) developed by Davis in 1989 as a theoretical framework for investigating CHNs’ acceptance of the IWSS in daily practice [17]. The TAM suggests that it is possible to show how the characteristics of technological devices influence their usage and users attitudes, by identifying the particular beliefs that come into play when they are used. Two theoretical constructs – perceived usefulness (PU) and perceived ease of use (PEOU) – are fundamental determinants of a new system’s use. PU is defined as “the degree to which a person believes that using a particular system would enhance his or her job performance”, [17] and PEOU refers to “the degree to which a person believes that using a particular system would be free of effort” [17].

## Study Aims

This study involved a secondary analysis of the qualitative data collected during of a pilot RCT. We aimed to explore the perceived acceptability (PU and PEOU) of an IWSS in the daily practice of referent CHNs caring for home-dwelling older adult patients. This innovative technology is designed to rapidly detect health issues such as falls, acute infections, delirium or immobilization, with the aim of preventing or avoiding hospitalization or emergency department visits.

## METHODS AND MATERIALS

### Study Design

This study reports on a secondary analysis of data collected during a previously conducted pilot RCT among home-dwelling older adults. Data came from individual interviews exploring the components of the IWSS’s acceptability after three-month, pilot RCTs conducted between August 1, 2014, and March 31, 2015. The trials involved using an IWSS intervention, as part of a tele-health protocol in the daily practice of CHNs, to rapidly detect health issues among older adults receiving home healthcare.

### Setting and Participants

The original RCT pilot study was conducted in a home healthcare service district in the French-speaking part of Canton Valais, Switzerland. The home healthcare center offers different services, such as nursing care (follow-up treatments, ADL support, and health assessment), daily meals, domestic cleaning, and social and administrative support. All the referent CHNs of older patients with an IWSS installed in their homes for the intervention participated in the study. The study was approved by the Canton’s human research ethics committee in June 2014 (CCVEM 020/14).

### Sample and Recruitment of Participants During the Pilot RCT

The principal investigator (PI-HV) and the study nurse (SN-CC) attempted to recruit 99 home-dwelling older adult participants, of whom 68 gave their written informed consent. These were assigned to either the experimental group (EG; n = 34) or the control group (CG; n = 34) using opaque sealed envelopes. A total of 57 participants completed the study: 29 in the EG and 28 in the CG. The PI, SN, and district home healthcare service were aware of the group allocations because the technological interventions needed to be prepared and adapted to the participants in the EG. EG participants received the IWSS intervention in addition to “usual care” interventions planned by the district home healthcare center. Participants in the CG received only the identical “usual care.”

After a presentation in collaboration with community healthcare supervisors, all eligible CHNs were invited to participate in the study. Eligibility criteria were: i) to have been a referent front-line CHN for at least three months; ii) to be at least a half-time (50%) employee during the study period.

### Intelligent Wireless Sensor System Intervention

Prior to the intervention, the IWSS study protocol and the management of alerts were explained to the referent CHNs. They participated in a two-hour training session on how to use the IWSS software. Thirty-four home-dwelling older adults in the Experimental Group [EG] had their IWSS installed within 72 hours of their allocation [18]. Prior to a three-month RCT with the IWSS, a two-week assessment period evaluated the participating older adult’s patterns of behavior. Individual alert thresholds for use during the RCT were chosen through discussions between the older adult (if possible), the home healthcare center, CHNs and informal caregivers.

The IWSS intervention continuously recorded home-dwelling older adults’ movements and changes in activity in strategic places in their living space: living room, bedroom, toilet, time spent in bed, and the number of times the refrigerator was opened. Using mobile technologies, the data collected in homes were sent to the IWSS data management center for analysis [19]. A data algorithm analyzed and detected changes in behavior patterns (>20% deviation) based on the participating home-dwelling older adults’ behavior patterns over the two previous weeks. Once a day, the system sent an alert to the referent CHN depending on the nature of participants’ changing behavior patterns, such as falls, acute infection suggested by more frequent visits to the toilet, or shorter stays in bed (See Supplementary File 1 for detailed information). The CHNs received alerts by SMS, email, or smartphone applications in a cascade. First of all, the IWSS sent an initial alert to the CHN by SMS, followed by an alert by email, and then a smartphone reminder. The CHN could then access the IWSS smart application dashboard to discover the nature of the change in movements or activity patterns.

### Study Outcomes

The study’s main outcomes were measured using: i) the PU and PEOU of the IWSS among CHNs, using self-administered questionnaires and focus group discussions; ii) the number of alerts transmitted during the study period; and iii) an assessment of the pertinence of the IWSS alerts for CHNs in their daily practice.

### Assessment of Acceptability Among CHNs

Data about acceptability were collected throughout the study. These came from personal notes written by the principal investigator and study nurse, and the answers from the questionnaires distributed to the referent CHNs after their experience of using the IWSS in daily practice with their patients. In the absence of a validated questionnaire on the acceptability of the IWSS, a questionnaire was developed based on the scientific publications by Davis [17], Venkatesh *et al.* [20], and Peek *et al.* [21]. It assessed the usefulness, ease of use, and perceptions of the use of the IWSS technology, using a 5-point Likert scale scoring from 0 “unacceptable/completely unsatisfactory” to 4 “extremely acceptable/very satisfactory”. Two open-ended questions, based on the critical incident technique developed by Flanagan [22, 23], allowed the CHNs to express their opinions and describe their positive and negative experiences of the IWSS. The alerts generated by the IWSS, together with their relevance in relation to deteriorating clinical situations, were transmitted to the IWSS data management center [19]. Finally, the referent CHNs who participated in the study with their patients were invited to participate in two focus group in order to make suggestions about the use, usefulness, and implementation strategy for IWSSs in daily practice.

### Data Analysis

The acceptability of using the IWSS, perceptions of the relevance or usefulness of the alerts that it generated, and its ease of use were all analyzed *via* returned questionnaires and the data collected from the transcripts of the audio-recordings of CHN focus groups. To obtain a clear idea of end-users’ satisfaction with the IWSS, the five-point Likert scale questionnaire answers were dichotomized: scores of 3 or 4 were recoded as “satisfied”; scores of 0 to 2 were recoded as “dissatisfied”. Satisfaction rates and the alerts generated by the IWSS were analyzed and descriptive statistics were performed using version 22 of the IBM-Statistical Package for Social Sciences (IBM-SPSS^®^) [24]. A statistical significance level was established at *p* = 0.05, and all tests were two-tailed.

Qualitative data were summarized using content analysis [25]. The information collected during the focus groups and open-ended questions was processed as follows: 1) for the group interviews, summaries were audio-recorded in a digital format; 2) the interviewer analyzed content from the focus group and the open-ended questions using NVivo 11 for Windows ^®^ [26] to identify positive, negative, or neutral events; 3) critical incidents were identified, classified, and organized by event type. The hierarchical categorization procedure followed the Flanagan method [22, 23]. During this classification into event types, the taxonomy of specific attitudes and the creation of new categories developed continuously, as Flanagan proposed, so that every incident was classified according to the specificities of the processes and participants involved [23].

## RESULTS

### Sociodemographic and Professional Characteristics

A total of 17 referent, front-line CHNs agreed to participate and completed the study. The sample was composed of two male and 15 female registered community healthcare nurses, with an average age of 26.4 years old, and who had been practicing home healthcare for 5.1 years on average. To ensure that the ethical committee’s criteria that no participants should be recognizable in the study findings, no further sociodemographic or professional characteristics are presented.

### Acceptability of the IWSS Interventions

Table **1** presents a summary of the CHNs perceived rates of satisfaction taken from the acceptability questionnaire. In general, acceptability rates among CHNs were moderate to low. Perceived ease of use for the IWSS received the lowest rate of satisfaction, whereas the understandability of the IWSS software scored highest.

### Usefulness of the IWSS Technology

The usefulness of the IWSS was rated using the number of alerts generated that were judged to have been pertinent. The IWSS technology generated and sent 638 alerts to the referent CHNs, of which they validated 608. Of these 40 (7%) were technical errors, 337 (55.4%) were judged to have been irrelevant for clinical evaluation, 94 (15.5%) required further investigation (an extra telephone call or home visit), and 137 (22.5%) were directly useful for the CHNs’ clinical practice. The IWSS alerts were able to inform CHNs, for example, of the time patients spent in bed or up, or the frequency of visits to the toilet, useful for detecting insomnia, falls, or a urinary tract infection. During the focus groups, CHNs estimated that the IWSS had “*not brought real gains to patient care among patients with a stable health condition*”. Fundamentally, the CHNs considered that IWSS technology was irrelevant for daily clinical preventive care for stable older patients. As already indicated, an IWSS alert was given when changes in home-dwelling older patients’ patterns of behavior increased or decreased by more than 20%. Those 20% changes were based on participants’ stable habits and behavior, however, some significant changes in behavior had nothing to do with health issues, such as falling asleep in the living room at night or going out with a friend. These were potential confounders for the clinical and practical monitoring of patients at risk. Furthermore, in daily practice, the elevated amount of non-relevant data generated by the IWSS made alerts difficult to interpret or integrate and reinforced the perception of clinical irrelevance. One CHN spoke of how *“we received many false alarms about changes in mobility patterns, … even though she went out to walk her dog every day, even though she’d gone out to visit her GP, we really checked up on her every time and the alerts were irrelevant”*.

Based on their professional experiences and bearing in mind the dramatic consequences of undetected falls at home, CHNs considered fall detection to be the most important issue in home healthcare monitoring. Unfortunately, the complexity of use of the IWSS added no value as a safer way to monitor and detect falls. Some information generated by the IWSS was not always appropriately health-related, and data on the number of refrigerator openings does not guarantee regular nutrition or imply behavior changes provoked by an unexpected visit from a formal or informal care provider. One experienced CHN reported that, *“One practical difficulty was adapting alert settings […]. So, there are moments when we said, ‘We didn’t set our alert indicators correctly, I think we should widen them.’ And the impact of changing alerts was actually difficult to measure. We had a lot of difficulties evaluating that. If I set the indicators to there, what impact will that have in terms of detection?”*

A major problem with motion detection was the presence of visitors, as it generated inappropriate interpretations of older patients’ risky behaviors. This caused supplementary telephone calls or home visits and raised tension between the referent CHNs and the older patients and their informal caregivers. Participating CHNs felt that the IWSS was intrusive and that older adults might feel that they were being permanently watched. One CHN described the following example: *“It’s true that I called a lady one time because we got an alert: she’d been going out less over the last three days; there’d been fewer signs of mobility from her front door and I called her up to be on the safe side. I asked her: ‘How are you? You’ve been going out less these days?’ And she said to me, ‘Yes, I’ve been out less. What’s worrying you?’ It was like, ‘What’s it got to do with you?”*

“*For me, the only type of case where the IWSS would be useful, would be with an Alzheimer patient who doesn’t know that he has to push his button anymore; with a person who doesn’t know how to ask for help*.”

Some alerts were not precise enough to permit a clinical interpretation, so CHNs were forced to call the data center or the older adult in order to clarify and understand the data generated by the IWSS. This resulted in repeated contact with the older adults and their relatives, sometimes inducing unnecessary worry about health and behavior; it also gave CHNs extra work. Indeed, only one CHN considered the data generated by the IWSS to be useful for clinical practice (*e.g.*, for adjusting nursing care plans) without having an impact on workload. CHNs considered the IWSS technology more useful for cognitively impaired older adults living at home. The following reflection demonstrated this.

Graphs of the patients’ day-time and night-time activities described their behavior patterns well and the CHNs considered these very useful. However, they viewed the toilet and refrigerator sensors, and the abundance of alerts which they sent out, to be irrelevant, merely disturbing and increasing their workload. Furthermore, that data increased doubts about the patient's safety and health status and sometimes generated unnecessary nursing interventions.

### Perceived Ease of Use

#### Information and Training

The majority of the CHNs claimed not to be familiar with the IWSS technology. All of them perceived a lack of information and training about the IWSS intervention protocol, which had an impact on the system’s PEOU. The CHNs reported a need for training in the use of this technology, to prepare for its use in daily clinical practice. Despite the explanations and support offered by the Datacenter, CHNs were not clear about how to use this IWSS technology, including how to set the alert indicators and use the system’s data. They mentioned a lack of support for the use of the system in complex clinical situations. The following transcript illustrates this:

“*First, we were coached on how to get going. Some people came to explain things: it’s true, the alerts or rather the whole system isn’t easy to understand if you’re not using it all the time. Then suddenly you’re left alone, you’re a grown-up and you can verify things, but when you realize—once, twice, three times—that the alert is irrelevant and you can’t get an answer, you give up*.”

#### The Interface and the IWSS Proof of Concept

CHNs found that ease of use was negatively influenced by an imprecise software interface, especially for setting alert indicators. This resulted in under- or over-evaluations of older patients’ risks of a deteriorating health status, causing either irrelevant alerts or an absence of alerts in true situations of significantly deteriorating health status. The IWSS technology failed to detect two falls, probably because of a lack of suitably positioned sensors in very old houses with multiples small rooms. This led to questions about the adaptability of the IWSS system’s proof of concept in different housing. Finally, the CHNs did mention that the ability to receive IWSS alert messages on mobile phones or tablets was beneficial. However, poor mobile telephone connections in mountainous rural areas were a major barrier to both the system’s ease of use and usefulness, and to responding to emergencies if necessary.

#### Managing IWSS Alerts

Beside the effectiveness of the IWSS technology, home healthcare district supervisors were also interested in the possibility of healthcare insurance companies reimbursing such interventions; they considered this critical to integrating the technology into CHNs’ daily practice. In Switzerland, without recognition and reimbursement from the healthcare insurance system, the integration and use of IWSS alerts will not become available as a community healthcare nursing service. The telephone calls to older patients or their informal caregivers, in order to investigate alerts, consumed the CHNs’ time without any health insurance reimbursement. Furthermore, informal caregivers failed to manage some emergency alerts well due to their lack of knowledge and information on how to work the IWSS technology. This resulted in inappropriate responses to emergency alerts and provoked stress and tension between CHNs and some of the informal caregivers. The complexity of managing emergency alerts increased workloads, especially with older patients with neurocognitive impairment, who were mostly unable to remember or to explain the reason for their behavioral change. The following transcript illustrates this problem: *“So we could see that an emergency alert had been sent out, but we couldn’t know all the steps that had gone on behind this, whether family members had been asked to help or not.”*

### DISCUSSION

The present study focused on the acceptability of innovative technology with which CHNs could monitor behavior changes of home-dwelling older patients, together with its perceived usefulness and perceived ease of use.

The present findings highlight that simply inventing a technology will not lead to its effective use; it will need a well-organized implementation and information strategy, and this confirms the recent study findings among healthcare providers by Peek *et al.* [27]. To address this study’s situation, and surely many others in the future, technology providers should tailor technologies to the specific needs of the community-dwelling older adults and their informal and formal caregivers. They will also need to adopt well-developed implementation strategies that fit in with the daily working practices of nurses in order to support this type of service delivery on a large scale. Nevertheless, interest in introducing innovative technologies for older adults in home healthcare situations is driven by multiple converging trends: the rapid pace of technological development; the unprecedented growth of aging populations worldwide who wish to remain at home as long as possible; the increase in the number and survival of people with disability; the growing and potentially unsustainable costs to government agencies of caring for so many older adults; and business and industry desires for profits [28]. These trends are contributing to the conviction that highly efficient technologies can play an important role in enhancing autonomy and quality of life for home-dwelling older adults, potentially reducing the individual and societal costs of caring for them. This corroborates the statements of Rialle that innovative technologies have a real potential to remain longer declining home dwelling older adults at their place [29]. Additionally, recent studies have documented the effectiveness of innovative e-health and Nanotechnology, Biotechnology, Information technology and Cognitive science in the management of chronic diseases like heart failure or type II diabetes in older patients [30, 31]. There is significant potential for combining IWSS and e-health technologies to support patients with chronic diseases in both community and primary care settings, but the potential downsides of adopting new technologies should not be forgotten, and nor should patient-identified needs and concerns.

Our findings highlighted that dealing with alert indicators was problematic for nurses, due to a lack of information about proof of concept and using the IWSS software. This corroborated recent research findings about using information and communication technologies to take care of older adults with dementia, which suggested that healthcare providers should be directly involved in the conceptualization of innovative technology as well as developing its usefulness and ease of use [32]. This is also an issue of professional accountability for the quality of care and patient safety. PU and PEOU seem to be pertinent variables with which to assess the implementation status and acceptability of innovative technologies among CHNs, confirming the findings of Holden and Ben-Tzion [33].

### Implications for CHNs’ Clinical Practice

Although CHNs reported that some irrelevant data generated by the IWSS increased the number of unnecessary nursing interventions for home-dwelling older patients, the majority declared that the technology was very useful for detecting falls and rapidly generating pertinent interventions to deal with them, especially for patients who were socially isolated or cognitively impaired. However, the same technology was deemed inadequate for safely reducing the number of visits by CHNs to older adults. CHNs did not want health insurance companies to be able to impose IWSS technology for remotely monitoring the risks of deteriorating acute health conditions among frail older adults; they felt this might result in fewer home visits to those with poor support networks, a lack of human contact, social isolation, and loneliness.

### Implications for Research and Practice

As noted throughout the present study, CHNs are in a position to lead the introduction of innovative technologies into their daily practice and clinical decision-making. However, implementation is a challenge. Another challenge for researchers is the frequent inability to provide sufficient proof of technologies effectiveness over usual care. Most innovative-technology implementation studies are observational or quasi-experimental because the technology is, for ethical and pragmatic reasons, delivered to older patients without randomization and comparison with a control or usual care group. Conducting research rigorously is by definition challenging, and research into innovative technologies should also respect high standards: confounding factors should be addressed to compensate for a lack of randomization.

IWSS technology is an excellent example of a decision support mechanism for CHN interventions. Thus, in its evaluation, it is important to monitor: (a) how consistently the intervention is applied to understand the amount of exposure to the advice; and (b) any other interventions occurring simultaneously that might affect outcomes. However, an IWSS intervention should be integrated into the existing decision support mechanisms aiding the daily practice of CHNs. Unfortunately, there are no one-size-fits-all interventions, and healthcare providers must never lose sight of older patients’ individual needs and instances in which decision support is not applicable [34]. Our findings showed that CHNs estimated that the IWSS system “*did not bring real gains to patient care*” for older patients in stable health. In other words, CHNs considered that the IWSS intervention was “*not useful*” as a preventive clinical support mechanism for home-dwelling older patients—but that is its primary purpose.

The present study showed that IWSS technology was not always easy to use and had low-to-moderate acceptability during focus group discussions with the CHNs involved. Despite the importance CHNs give to the early detection of health issues among home-dwelling older adults, IWSS technology only got moderate scores for usefulness, as revealed by one CHN’s statement that, *“The IWSS wasn’t able to detect health issues, because there were too many system hiccups providing non-relevant information […], inducing more questions than answers.”*

The IWSS intervention was meant to prevent avoidable hospitalization and allow older adults to remain in their homes, however it sometimes failed. On the one hand, CHNs reported that the IWSS generated several inappropriate preventive alerts: *“This information, when sent repeatedly, does not allow us to evaluate real clinical care needs, so we quickly stopped using it.”* On the other hand emergency alerts that were transferred solely to informal caregivers failed to result in appropriate, well-organized care activities with follow-up.

Intruding on the privacy of home-dwelling older adults in order to validate alert messages was an important barrier to the ease of use of the IWSS intervention for CHNs. Moreover, some of the families included in the study refused to continue their participation in the study; they feared losing family privacy, disclosing family conflicts, or over-intrusive assessments—findings also documented by Halstall *et al.* [32] and Lorenzen-Hubber [35]. The IWSS technology also demonstrated multiple unexpected technical failures, which caused CHNs to doubt its usefulness and applicability to the safety of older patients at risk of falls. In interviews about technology and healthy aging at home, CHNs who had not been involved in the IWSS trial revealed that their perceptions of innovative technologies were significant determinants of their intention to use them and probably influenced their perceptions negatively. Moreover, CHNs’ positive and negative attitudes lead to corresponding higher and lower usage intentions. Technologies should be developed and implemented in close collaboration with the end-users so that their perceptions are considered throughout the process; this should add value and yield better outcomes. Although promising results about using innovative technologies to assess and monitor health issues among home-dwelling older adults have indeed been documented, direct observations and clinical assessments still seem the most appropriate methods for assessing changes in autonomy [36, 37].

Finally, effective, practical innovative technologies certainly have a role to play in the future optimization of healthcare workforces, infrastructure, and financial resources. Hence, innovative technologies such as the IWSS should be financially affordable for all older adults, independently of their income or health status. Nonetheless, healthcare systems, relatives and patients should not be able to impose the use of these technologies on healthcare professionals.

### Study Limitations

This study had various limitations. The first concerns its selective exploration of two components of the acceptability concept. Another limit was the use of non-validated and auto-constructed, non-exhaustive questionnaire exploring satisfaction and acceptability. In addition to the relatively small sample, it seems important to mention that data were collected *via* a single district home healthcare center, as this raises questions as to the transferability of our findings: extending them to other technologies or regions should be done with great caution. Finally, our small study population of CHNs was particularly unfamiliar with innovative technologies, thus transferring and generalizing our findings to other CHNs is impossible.

## CONCLUSION

Community healthcare nurses' roles with home-dwelling older patients have evolved, as have their roles in the use of technology to detect health issues and improve healthcare delivery. In an effort to address acute health issues rapidly, increase patient safety, and reduce healthcare-associated costs, CHNs are being challenged to incorporate technology into their daily nursing practice. Despite, the low-to-moderate acceptability of an intelligent wireless senor-system intervention in their daily clinical practice, CHNs are quite convinced of the potential for future innovative technologies to help older adults remain in their homes, optimize patient safety, and contain costs.

Our findings also demonstrated that technology providers should invest sufficient time to understand CHNs daily practice and work processes and develop well-organized implementation and information strategies.

## Figures and Tables

**Table 1:** Acceptability of IWSS technology among Community Healthcare Nurses (n = 17).

**Items**	**% of positive responses from CHNs (n = 17)**
**Satisfaction with the use of the IWSS** - Compared to presentation of the IWSS that was made to you, were you satisfied with the use of the technology? - How would you evaluate the usefulness of the IWSS? Did it help you? - In your opinion, did the IWSS become an indispensable part of your daily activity?	6% (n = 1)6% (n = 1)(n = 0)*
**Installation of the IWSS** - Was the length of the assessment period prior to the installation of IWSS acceptable? - Concerning the previous interview, was the content of the previous interview acceptable? - Was that the duration of sensor placement acceptable? - Was setting the alert indicators easy to do?	50% (n = 8) 31% (n = 5) 50% (n = 8) 59% (n = 10)
**Adherence** - Did you have difficulty understanding how to use the IWSS’s software? - Could you integrate data from the IWSS into your patients’ care management?	59% (n = 10) 6% (n = 1)
**Credibility – Ease of use – Safety** - Was the IWSS interface easy to use? - Was the IWSS a reliable and appropriate one with which to ensure the safety of home-dwelling older patients? - Were the alert messages useful for adapting your patients’ care? - Was the IWSS better than you expected? - Was the IWSS easy to integrate with other technologies used in your daily practice? - The IWSS did not overload daily planned care and nursing activities? - Did the IWSS provide you with useful information that is otherwise inaccessible?	29% (n = 5) 18% (n = 3) 0% (n = 0)* 0% (n = 0)* 6% (n = 1) 6% (n = 1) 6% (n = 1)
**Information collected by the IWSS available for CHN (638 alerts generated)** - Alerts judged to have been useful (n = 608 validated) - Alerts which needed further investigation - Alerts judged to have been irrelevant - Alerts judged to have been technical errors	23% (n = 137) 16% (n = 94) 56% (n = 337) 7% (n = 40)

Note*None of the participating CHNs considered the IWSS provided useful, credible information.
